# Retrograde intramedullary K-wire fixation of trapezoid dislocation: a case report

**DOI:** 10.1080/23320885.2025.2594249

**Published:** 2025-11-23

**Authors:** Chih-Hsun Chang, Chin-Hsien Wu, Hui-Kuang Huang

**Affiliations:** a Department of Orthopedics, Ditmanson Medical Foundation Chiayi Christian Hospital , Chiayi, Taiwan; b Department of Biomedical Engineering, National Cheng Kung University, Tainan, Taiwan; c Department of Orthopedics, E-Da Hospital, I-Shou University, Kaohsiung, Taiwan; d School of Medicine, College of Medicine, I-Shou University, Kaohsiung, Taiwan; e Department of Food Nutrition, Chung Hwa University of Medical Technology, Tainan, Taiwan

**Keywords:** Carpal, dislocation, carpometacarpal, intramedullary K-wire, trapezoid

## Abstract

Trapezoid dislocations are extremely uncommon injuries because of the strong intercarpal and carpometacarpal ligaments that provide greater stability to the trapezoid–metacarpal joint compared with the more ulnar carpometacarpal joints. We report a case of trapezoid dislocation, emphasizing the radiographic features essential for diagnosis and the fixation strategy used for management. A 50-year-old woman sustained a sliding fall while riding a scooter, resulting in a trapezoid dislocation from the carpometacarpal joint accompanied by a fracture at the base of the right third metacarpal. The injury was managed with retrograde intramedullary K-wire transfixation of the second metacarpal-trapezoid joint combined with a spanning plate across the thrid metacarpal-capitate joint. The spanning plate maintained the length of the third metacarpal relative to the capitate, thereby indirectly restoring the anatomical alignment of the second metacarpal and stabilizing the reduced trapezoid. The retrograde intramedullary K-wire was easily positioned so that its tip did not extend beyond the trapezoid, thus preventing potential irritation of surrounding vital structures that might result from an obliquely placed K-wire. The ‘missing carpal sign’ serves as an important radiographic clue suggestive of trapezoid dislocation. Given the strong surrounding interosseous ligaments, it is important to recognize the possible occurrence of trapezoid dislocation in association with fractures or dislocations involving structures adjacent to the trapezoid. We present a retrograde intramedullary K-wire fixation technique for stabilizing the trapezoid–metacarpal joint, which can be easily and effectively applied after anatomical reduction of the trapezoid.

## Introduction

Trapezoid dislocations are extremely uncommon injuries because of the strong intercarpal and carpometacarpal (CMC) ligaments that provide greater stability to the trapezoid-metacarpal joint compared with the more ulnar CMC joints. The mechanism of injury is typically related to axial compression and bending forces transmitted through the base of the second metacarpal [1–4]. We report a case of trapezoid dislocation associated with the third metacarpal base fracture, along with the fixation strategy used for its management. A review of the relevant literature was also performed to enhance understanding of this rare injury.

## Case presentation

**Figure 1.**
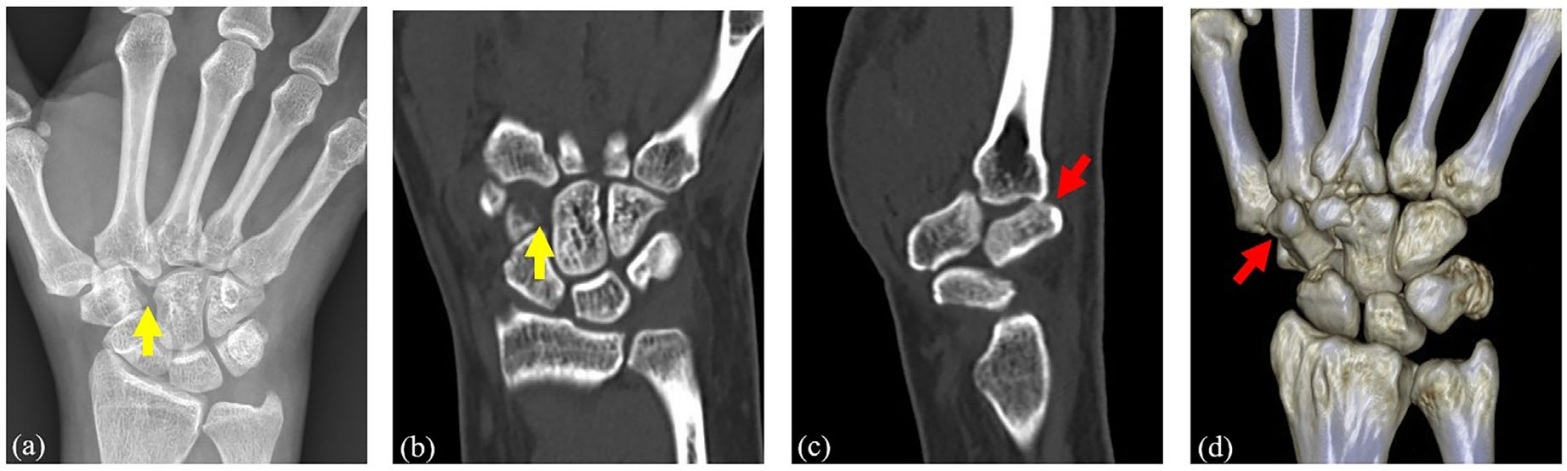
(a, b) Radiographs and CT images show a “missing carpal sign” (yellow arrow). (c, d) CT images show a trapezoid dislocation out of the carpometacarpal joint (red arrow).

A 50-year-old right-hand-dominant woman sustained a sliding fall while riding a scooter. She reported that she had been holding onto the scooter’s handlebars during the fall. She presented to the emergency department with an injury to her right hand. Initial radiographs identified only a fracture at the base of the third metacarpal. However, due to disproportionate swelling, she was referred to the hand clinic for further evaluation and treatment. Upon review of her radiographs, a ‘missing carpal sign’ was noted, showing an abnormally widened space at the trapezoid–capitate junction in addition to the third metacarpal base fracture. Computed tomography (CT) revealed not only a fracture at the base of the third metacarpal but also an empty space proximal to the base of the second metacarpal on coronal images, while sagittal images demonstrated a dorsal dislocation of the trapezoid head in relation to the second metacarpal (Figure 1).

Surgery was scheduled and performed 7 days after the injury under general anesthesia with the arm placed on a hand table and a pneumatic tourniquet applied. A longitudinal incision was made between the second and third metacarpals, extending proximally to the midcarpal level. The trapezoid was easily reduced by applying palmar pressure while providing traction to the index finger.

For the third metacarpal base fracture, a spanning plate was applied from the capitate to the third metacarpal to maintain metacarpal length. We used a 1.3-mm Acumed Hand Fracture System T-plate (Acumed, Hillsboro, Oregon). Two holes were trimmed from the straight end of the original 10-hole T-plate (59.9 mm), after which the plate was inverted for fixation. This configuration allowed the three 2.3-mm screws on the T-end to achieve purchase in the capitate, while the three screws on the straight end achieved secure purchase in the third metacarpal shaft, with the middle two screw holes intentionally skipped to avoid the fracture site. Restoration of the third metacarpal length is crucial for keeping the second metacarpal at its proper level, given that the interosseous ligament between the second and third metacarpals remains intact. With the correct alignment restored, the reduced trapezoid was subsequently stabilized by transfixation to the second metacarpal.

The stability of the reduced trapezoid was confirmed, showing no tendency to displace during flexion of the second metacarpal. A 1.0-mm K-wire was then inserted from the radial corner of the second metacarpal head in a retrograde intramedullary manner to transfix the trapezoid–metacarpal joint.

After surgery, digital motion rehabilitation was initiated. The K-wire was removed 6 weeks postoperatively, and the spanning plate was removed 3 months after surgery. Weight-bearing exercises were then started thereafter.

**Figure 2.**
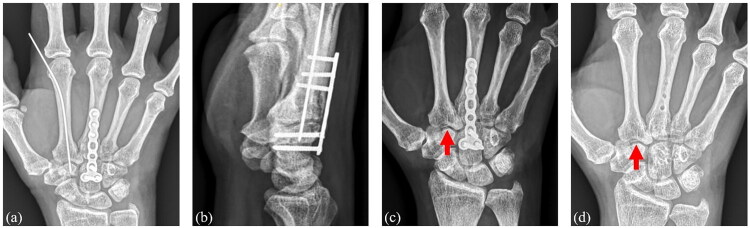
(a, b) Radiographs show retrograde intramedullary fixation of the reduced trapezoid–metacarpal joint and a spanning plate for the middle finger metacarpal base fracture at 1 month postoperatively. Radiographs at 3 months (c) and 1 year (d) postoperatively show a maintained reduction of the trapezoid–metacarpal joint (red arrow).

At the 1-year follow-up, radiographs demonstrated that the reduction of the trapezoid–metacarpal joint was well maintained, with no evidence of degenerative changes (Figure 2). The patient reported that the function of her right hand had fully recovered to the pre-injury level. The range of motion of the right and left wrists was 70° and 75° in flexion, and 75° and 75° in extension, respectively. Grip strength measured 24.3 kg on the right side and 23.9 kg on the left, while pinch strength was 5.6 and 5.2 kg, respectively. The shortened disabilities of the arm, shoulder and hand questionnaire (QuickDASH) was 11.4 at the 1-year follow up [5]. The patient declined further follow-up, being fully satisfied with her recovery.

## Discussion

Because trapezoid dislocation is an extremely rare injury, the diagnosis is often overlooked, with up to 30% of cases missed during initial evaluation [6]. This may be due to associated dislocations or fractures of adjacent structures that obscure the lesion, as well as unfamiliarity with the complex anatomic contour of the trapezoid region. In our case, the trapezoid dislocation was not identified on the initial radiographs obtained in the emergency department. However, certain radiographic clues suggested the presence of this injury. An increased space between the trapezoid and capitate, known as the ‘missing carpal sign’, was noted [2]. CT was useful in confirming the diagnosis, revealing an empty space proximal to the second metacarpal on the coronal view (Figure 1b).

We do not recommend using magnetic resonance imaging (MRI) as the initial evaluation tool instead of CT, because intraosseous bruising or ischemia may be present without necessarily progressing to true necrosis. The high sensitivity of MRI may lead to an overestimation of ischemic changes and potentially discourage attempts at open reduction, whereas avascular necrosis of the trapezoid after reduction has been rarely reported in the literature [7].

For surgical management of trapezoid dislocations, early surgical intervention is recommended whenever possible. In cases of delayed presentation of trapezoid dislocation, reduction and fixation should still be attempted. Although excision of the trapezoid or limited arthrodesis has been proposed as the salvage procedures for chronic dislocations, we would still recommend attempting an open reduction whenever feasible, as avascular necrosis after reduction has not been reported to our knowledge. Salvage procedures may be considered when symptomatic avascular necrosis is present or when adequate reduction cannot be achieved through an open approach [7–12].

The trapezoid is a wedge-shaped bone that articulates securely with the base of the second metacarpal. It is also firmly stabilized by adjacent carpal bones through strong interosseous ligaments. Because the volar ligaments are stronger than the dorsal ligaments, and the distal portion of the trapezoid has a larger dorsal than volar surface, the bone is predisposed to dorsal dislocation [13]. The mechanism of dorsal dislocation typically involves a bending force applied to the second metacarpal when the wrist is in slight flexion, followed by an axial loading force transmitted to the trapezoid, which acts as a lever and causes the bone to be extruded dorsally [4]. Because the radial CMC joints are more stable than the ulnar-sided CMC joints, any dislocation involving the radial CMC joints warrants careful consideration of the injury mechanism and the direction of force transmission.

There can be several variations of trapezoid–metacarpal joint dislocation. In some cases, the second metacarpal may be dislocated, most commonly dorsally, while the trapezoid remains in place. This pattern is usually easier to identify on a lateral wrist radiograph [1]. Alternatively, the trapezoid itself may dislocate proximally at the scaphotrapeziotrapezoid (STT) joint [1], or the injury may involve force transmission through the capitometacarpal joint, resulting in dislocation of the third metacarpal from the capitate while the trapezoid–metacarpal and second–third intermetacarpal joints remain intact [3]. In other instances, the force may extend further ulnarly, leading to additional CMC joint dislocations [14].

Axial loading forces can also cause trapezoid fractures, most often presenting as coronal plane fractures with either the dorsal or volar fragment displaced [3,15]. Regarding dislocation direction, volar dislocation is not uncommon, although dorsal subluxation accounts for approximately two-thirds of reported trapezoid dislocations [3,16].

Several associated injuries have been reported, including fractures or dislocations of the metacarpals and CMC joints, various intercarpal dislocations [3,17,18], and even Galeazzi fracture–dislocations [19]. Rare complications such as attritional rupture of the index flexor tendons [20], and acute carpal tunnel syndrome secondary to trapezoid dislocation have also been described [16].

Although no single, definitive mechanism of force transmission has been established to serve as a predictive indicator for trapezoid dislocation, we suggest that meticulous physical examination and detailed injury history, combined with careful review of radiographs and CT imaging, can significantly reduce the likelihood of missed diagnosis.

For the treatment of trapezoid dislocation, early reduction is recommended, as avascular necrosis may occur if the bone’s circulation is compromised, although this complication is rarely reported in the literature [21]. Closed reduction can be attempted in cases of dorsal dislocation; however, if this approach fails, open reduction through a dorsal approach should be performed. K-wire fixation of the trapezoid–metacarpal joint is commonly used, with the wires typically retained for 4 to 6 weeks before removal [3,7]. Although immediate limited carpal fusion has been proposed by some authors [17], we suggest reserving fusion as a salvage procedure for cases with persistent instability or degenerative changes detected during follow-up.

For K-wire fixation of the trapezoid–metacarpal joint after reduction of the trapezoid, we proposed a retrograde intramedullary technique. A 1.0-mm K-wire was inserted from the radial corner of the second metacarpal head in a retrograde manner, passing through the intramedullary canal and exiting the base cortex of the second metacarpal into the trapezoid, thereby transfixing the trapezoid–metacarpal joint. This method offers several advantages.

First, intramedullary transfixation provides a more centralized and stable K-wire trajectory (Figure 2a, b). In most previously reported techniques, the K-wire is inserted obliquely from the metacarpal base into the trapezoid or across to the adjacent carpal bones [3]. However, with oblique insertion, it is often difficult to confirm how far the K-wire extends volarly. If the wire inadvertently penetrates the volar cortex of the trapezoid, it may subsequently migrate volarly during the fixation period, potentially injuring the volar tendons or neurovascular structures.

In contrast, with our retrograde intramedullary approach, the K-wire path can be easily visualized on a standard anteroposterior view, allowing clear assessment of its depth within the trapezoid. Even if the K-wire inadvertently penetrates the proximal cortex of the trapezoid, the risk of damage would be limited to the scaphoid head, which is considerably less hazardous than injury to the volar soft tissues.

Another advantage of this technique is that, whether the K-wire is buried or left exposed, its entry point on the radial side of the metacarpal head poses no risk to the extensor tendons. In contrast, obliquely positioned K-wires at the carpometacarpal joint may lie close to the extensor tendons, increasing the risk of complications such as tendon attrition, tenosynovitis, or infection.

## Conclusion

Trapezoid dislocations are exceedingly rare injuries, often overlooked due to their subtle radiographic appearance. In the present case, several key points can be emphasized. First, the ‘missing carpal sign’ serves as an important radiographic clue suggestive of trapezoid dislocation. Second, awareness of the possible occurrence of trapezoid dislocation is crucial when evaluating fractures or dislocations involving structures adjacent to the trapezoid–metacarpal joint. Finally, we present a retrograde intramedullary K-wire fixation technique for stabilizing the trapezoid–metacarpal joint, which can be effectively applied once the surrounding structures have been anatomically restored and stabilized.
